# Age- and sex-specific transcriptomic changes drive the prothrombotic potential of megakaryocytes

**DOI:** 10.1186/s40364-025-00830-x

**Published:** 2025-10-14

**Authors:** Pratintip Lee, Carolina Balbi, Meret S. Allemann, Adhideb Ghosh, Tongtong Wang, Marco Bacigaluppi, Alexander Akhmedov, Giovanni G. Camici, Thomas F. Lüscher, Adriano Fontana, César Nombela-Arrieta, Christian Wolfrum, Seyed Soheil Saeedi Saravi, Jürg H. Beer

**Affiliations:** 1 Center for Molecular Cardiology, University of Zurich, Wagistrasse 12, Schlieren, 8952 Switzerland; 2 Department of Medicine, Cantonal Hospital Baden, Baden, Switzerland; 3 Laboratory of Translational Nutrition Biology, Institute of Food, Nutrition and Health, Department of Health Sciences and Technology ETH Zurich, Schwerzenbach, Switzerland; 4 Neuroimmunology Unit, Institute of Experimental Neurology, Division of Neuroscience, IRCCS San Raffaele Hospital and Vita-Salute San Raffaele University, Milan, Italy; 5 Neurology Department, IRCCS San Raffaele Hospital, Milan, Italy; 6 Department of Research and Education, University Hospital Zurich, Zurich, Switzerland; 7 Royal Brompton & Harefield Hospitals, Imperial College and King’s College, London, UK; 8 Institute of Experimental Immunology, Zurich, Switzerland; 9 Department of Medical Oncology and Hematology, University Hospital Zurich, Zurich, Switzerland; 10 Center for Translational and Experimental Cardiology, Department of Cardiology, University Hospital Zurich, University of Zurich, Wagistrasse 12, Schlieren, 8952 Switzerland; 11 University Heart Center, Department of Cardiology, University Hospital Zurich, Zurich, Switzerland

**Keywords:** Aging, Megakaryocytes, Thrombosis, Inflammation, Single-cell Transcriptomics, Mitochondria, Sex

## Abstract

Aging is associated with enhanced platelet activation and inflammatory responses, contributing to an increased risk of thrombotic and cardiovascular events. However, how megakaryocytes (Mk) change with age or between sexes is still unclear.

We performed single-cell RNA sequencing to profile bone marrow from young (3-month-old) and old (> 24-month-old) C57BL/6 mice, as well as from young and middle-aged human donors. In parallel, platelet activation was assessed by CD62P expression using flow cytometry in isolated and washed mouse platelets from young and old donors.

Single-cell clustering in mouse bone marrow revealed five distinct Mk subpopulations. The age-dependent shift was more pronounced for subpopulations associated with platelet activation (mMk3) and immune/inflammatory responses (mMk4). mMk3 cells were more abundant in females, whereas mMk4 predominated in males. An increased platelet activation phenotype in old mice was confirmed in both sexes by CD62P expression analyses. Differential gene expression analyses showed that aging alters gene expression patterns related to platelet activation and aggregation via receptor signaling, granule formation, and inflammation. Aged males exhibited enrichment in genes linked to mitochondrial function, oxidative stress, lipid metabolism and membrane dynamics, whereas aged females showed upregulation of serotonin receptor genes. Human transcriptomic data support our murine findings, exhibiting increased expression of genes involved in mitochondrial electron transport and immune signaling in middle-aged individuals.

Our study reveals a complex, sex-specific remodeling of Mk subpopulations in the aging organisms, characterized by distinct pro-thrombotic and pro-inflammatory transcriptional profiles. These observations suggest potential targets for developing age- and sex-tailored antithrombotic therapies.

**Supplementary Information:**

The online version contains supplementary material available at 10.1186/s40364-025-00830-x.

**To the Editor**

 Aging is linked to increased thromboembolic and cardiovascular events [1]. Dysregulated megakaryocytes (Mk) drive platelet hyperreactivity [2, 3]. Mk progenitors from aged mice expand 2.5-fold and accelerate platelet repopulation when transplanted into young recipients [4, 5]. Five Mk subpopulations, cycling, thrombopoiesis, immune-like and niche support, have been described, their age- and sex-specific dynamics remain unclear [6–8].

Using single-cell RNA sequencing, we generated an atlas of bone marrow (BM) Mk from young (3-month) and aged (24-month) male and female mice, and validated findings in human BM. We identified five subpopulations: cycling/polyploid (mMk1, mMk 2), thrombopoietic (mMk3), immune-like (mMk4), mMk5 and observed sex-specific age shifts (Fig. 1A-B, sFig.1A-C). mMk3 rose in aged males (8 → 10%), but more in females (16 → 26%), suggesting a female bias toward enhanced platelet production, consistent with report that females maintain higher platelet counts even after menopause [9] (Fig. 1 C). Conversely, immune-like mMk4 expanded in both sexes (male 2.4 → 9.7%, female 2 → 6.4%), indicating a shared immune adaptation with sex-modulated magnitude. mMk5, found in young males, declined with age, its heterogeneity limits functional interpretations (Fig. 1D). Both sexes showed higher CD62P surface expression on activated platelets, supporting increased activation and mMk3 expansion (Fig. 1E). We also observed a trend toward increased CD62P in resting platelets and enhanced thrombus formation on collagen (sFig.1H-I).

**Fig. 1:**
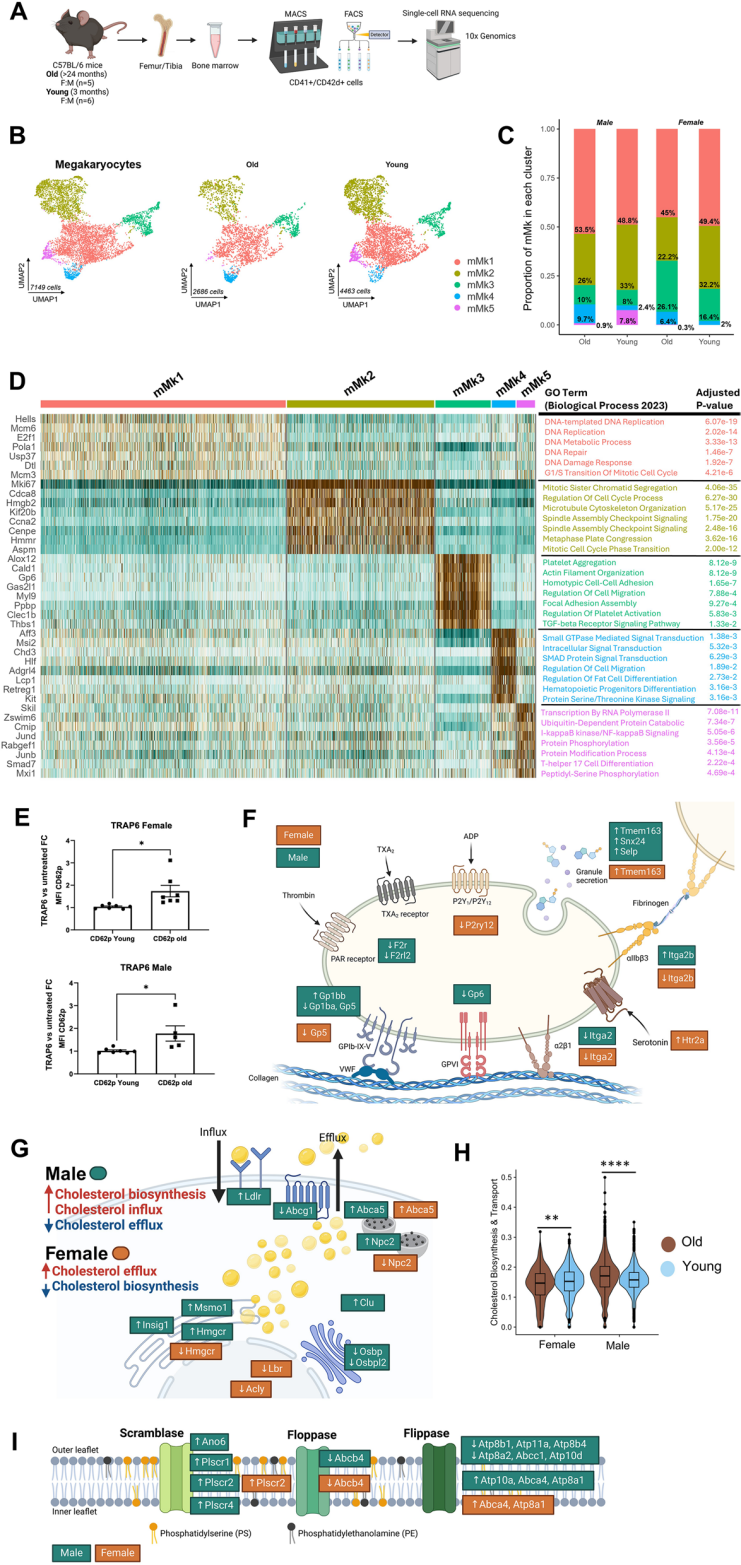
**A**) Schematic overview of mouse BM Mk isolation and scRNA sequencing workflow. Femur and tibia bone marrow cells from young (3 months, n=6) and old (>24 months, n=5) C57BL/6 mice of both sexes were harvested, enriched for CD41/CD42d cells using MACS and FACS, and subjected to scRNA sequencing using the 10x Genomics platform. **B**) UMAP of extracted and re-clustered Mk, colored by subpopulation (mMk1-mMk5; color key inset blue = downregulation, brown = upregulation). Insets display age-stratified embeddings (young, old). **C**) Proportion of each Mk subpopulation per sex and age group (bars show proportion and percentages of total Mk). Two proportion Z-tests with Bonferroni correction for multiple comparisons. Female: Significant after Bonferroni (α = 0.01): mMk2, mMk3, mMk4. Male: Significant after Bonferroni (α = 0.01): mMk1, mMk2, mMk4, mMk5. **D**) Heat-map of the top DEGs per subpopulation (rows = genes; columns = cells). Expression = z-score of log2(normalized counts + 1). GO terms (Biological Process 2023) with the lowest FDR are annotated on the right. **E**) Platelet activation assay. Fold change of TRAP6 compared to untreated of MFI ± IQR of CD62P on washed platelets after TRAP-6 stimulation (0.1 U ml-1, 15 min, 37 °C; Brilliant Violet 421 clone RB40.34). **F**) Diagram of Sex-divergent platelet-activation receptor genes. **G**) Cholesterol-biosynthesis/transport pathway diagram. **H**) Violin plot of the cholesterol-gene-set score in old mice (male and female). Wilcoxon rank-sum test with Bonferroni; P thresholds as below. **I**) Phospholipid-transport pathway diagram. • Statistics. *P* values: *< 0.05, **< 0.01, ***< 0.001, ****< 0.0001. • The lists of genes used to calculate the score are shown in Supplementary method. • Panel F, G, I Boxes highlight transcripts up and downregulated in males (green) or females (orange); all DEGs: FDR < 0.05.• Panel A created by Biorender

At the platelet receptor level, aged males and females exhibit partially reciprocal alterations (Fig. 1 F, sTable1). In males, the fibrinogen receptor subunit αIIb (Itga2b) was upregulated (1.39-fold, mMk2), while the PAR-1 receptor *F2r* was downregulated (0.7-fold, mMk1), suggesting altered aggregation dynamics. In females, ADP responsiveness fell (*P2ry12* 0.25-fold, mMk3), whereas the serotonin receptor *Htr2a* rose 8.75-fold (mMk3) (sFig.1D). These opposing signatures imply distinct aggregation mechanisms in both sexes.

Lipid metabolism and membrane dynamics diverged by sex. Aged male Mk upregulated cholesterol synthesis/uptake (*Hmgcr, Ldlr*) while downregulating efflux (*Abcg1*), suggesting intracellular cholesterol accumulation (Fig. 1G-H). Increased phospholipid scramblase (*Ano6/Tmem16f*) and reduced flippases (*Atp8b1*) indicate enhanced phosphatidylserine (PS) exposure and procoagulant vesicle shedding (Fig. 1I). In contrast, aged females downregulated cholesterol biosynthetic genes and maintained, or enhanced, efflux capacity (*Abca5*), with minimal flippase/scramblase changes. Increased PS exposure in aged male could amplify thrombin generation and microparticle release, contributing to male-biased prothrombotic phenotypes [10].

Mitochondria and oxidative-stress also diverged (Fig. 2A-B). Male Mk upregulated mitochondrial ribosomal proteins (*Mrpl/Mrps*), ETC subunits (Complexes I–IV) and ROS-detox genes (*Park7, Prdx5*), yet repressed glutathione- and peroxisome-based defenses (*Cat, Gsr*), suggesting heightened ATP production with ROS generation. Females downregulated both mitochondrial and antioxidant genes, consistent with reduced bioenergetic capacity but lower oxidative stress [11].

**Fig. 2:**
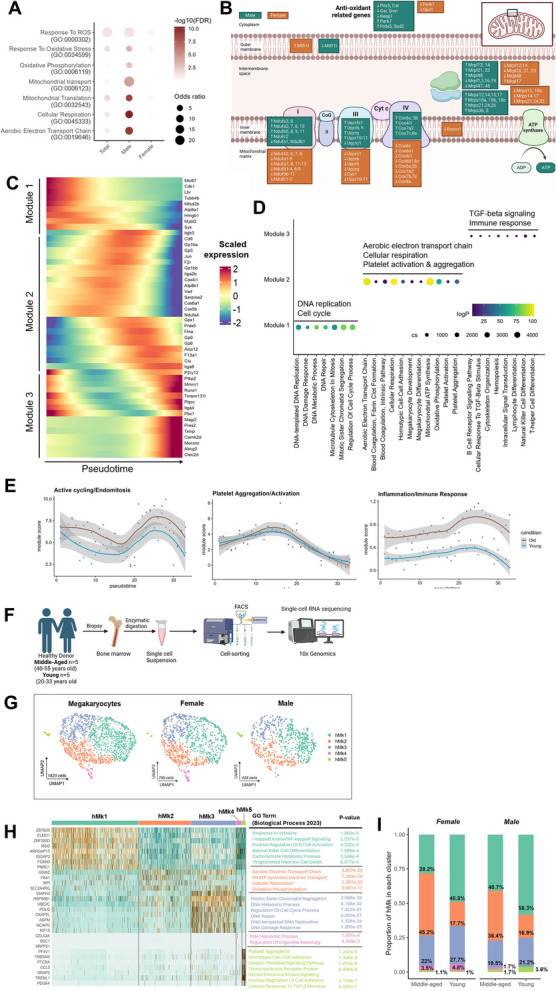
**A**) Dot-plot of enriched GO terms related to mitochondrial function among upregulated DEGs in old mice (total, male, female). Dot size = gene-ratio; color = –log10FDR. **B**) Pathway diagram summarizing mitochondrial DEGs; Boxes highlight transcripts up and downregulated in males (green) or females (orange); all DEGs: FDR < 0.05. **C**) Pseudotime modules of trajectory-wise DEGs in the Mk subpopulation. Only statistically significant genes from FDR adjusted *p*-values were selected. The genes were grouped into three modules. **D**) Dot-plot of the top GO biological process terms for each modules. Each bubble represents a pathway, with its x-axis position corresponding to the pathway name. Bubble color denotes the log10 transformed adjusted *p*-value (logP) from EnrichR (darker colors = more significant), and bubble size reflects the cumulative score (cs) for that pathway. LogP = log10(adjusted *p*-value); cs = EnrichR cumulative score. **E**) Smoothed module scores for active cyclic/endomitosis, platelet aggregation/activation and inflammation/immune response gene sets along Pseudotime in the old and young mice. The darker lines are the local regression result for individual time bins, with the gray shadow depicting the 95% CIs. The lists of genes used to calculate the score in I) are shown in Supplementary Method. Fig. 2A-E referred to murine data in Fig. 1. **F**) Schematic overview of the human BM scRNA-sequencing workflow. Biopsies from healthy donors (young = 5, 20–33 years; middle-aged = 5, 40–55 years) were enzymatically digested, isolated by FACS and profiled with10x Genomics. **G**) UMAP of extracted and re-clustered human Mk (hMk) colored by subpopulation; insets: male and female. **H**) Heat-map of the top DEGs per subpopulation(rows = genes; columns = cells). Expression = z-score of log2 (normalized counts + 1). GO terms (Biological Process 2023) with the lowest FDR are annotated on the right. **I**) Proportion of each hMk subpopulation per sex and age group (bars show proportion and percentages of total hMk) Two proportion Z-tests with Bonferroni correction for multiple comparisons. Female: Significant after Bonferroni: hMk1, hMk2. Male: Significant after Bonferroni: hMk1, hMk2. • Panel F created by Biorender

These sex-specific Mk programs were conserved in humans (Fig. 2F-G). In BM from 10 healthy individuals: 5 young adults (ages 20–33, 3F/2M) and 5 middle-aged adults (ages 40–55, 2F/3M) (sFig.2A), a mitochondrial ETC-enriched cluster expanded with age in both sexes (Fig. 2I, sFig.2B-C). Only females showed coordinated upregulation of mitochondrial-encoded and nuclear ETC genes. Female-specific up-regulation of PF4, together with upward trends in collagen- and serotonin receptor transcripts, mirrors murine receptor shifts (sTable2). Marker-overlap and differential-expression analysis supported cross-species conservation of age-related Mk patterns (sFig.1E-F).

Trajectory inference revealed a continuum from immature, cycling Mk to mature, pro-thrombotic, immune-skewed phenotypes (Fig. 2C-D). Cell-cycle gene scores show a biphasic pattern, peaking mid-trajectory, then declining, while platelet-activation genes rose to a mid-pseudotime apex; inflammatory rose at later stages (Fig. 2E). Cycling and inflammatory modules remain elevated in aged Mk, underscoring persistent age-related transcriptional biases (sFig.2G). This aligns with reports that aged platelets shift toward a thromboinflammatory profile with more platelet-leukocyte aggregates [12].

Strengths include a systematic, sex-specific comparison across age groups, uncovering distinct transcriptional signatures, and inclusion of human BM, providing a cross-species reference and suggesting conserved regulatory mechanisms. Limitations are: (i) lack of protein-level validation (partial support from flow cytometry and thrombosis assays); (ii) a cross-sectional design, pseudotime informs but cannot substitute for longitudinal maturation markers and functional assays; (iii) size-based sorting may underrepresent fully mature Mk; (iv) aging- and sex-associated candidate genes are identified but require mechanistic clarification.

Altogether, our data reveal a multidimensional, sex-specific reprogramming of aging Mk, encompassing subpopulation dynamics and functional pathways, consistent with the concept of aging-driven thrombo-inflammation, although causality requires confirmation. Within this framework, three mechanistic avenues emerge: lipid-membrane remodeling, receptor-repertoire shifts and mitochondrial/ROS adaptations. Overall, our findings provide a repository for mechanistic studies and support the development of sex-specific preventive and therapeutic strategies in the elderly.

## Supplementary Information

Supplementary Material 1.

Supplementary Material 2.

Supplementary Material 3.

Supplementary Material 4.

## Data Availability

The transcriptomics of young and old datasets generated and analysed during the current study are available in the GSE289417 repository, (https://www.ncbi.nlm.nih.gov/geo/query/acc.cgi?acc=GSE289417).

## Abbreviations

Mk: Megakaryocytes
BM: Bone marrow
PS: Phosphatidylserine
ETC: Electron Transport Chain
ROS: Reactive Oxygen Species
UMAP: Uniform Manifold Approximation and Projection
DEGs: Differentially expressed genes
GO: Gene-Ontology
FDR: False-Discovery Rate
MFI: Median Fluorescence Intensity
FC: Fold Change
IQR: Interquartile Range
hMk: Human Megakaryocytes
CD62P: P-selectin
ADP: Adenosine Diphosphate
5-HT: 5-Hydroxytryptamine
PAR-1: Protease-activated receptor-1
