# Supplementary material for: Age- and sex-specific transcriptomic changes drive the prothrombotic potential of megakaryocytes
Source: Biomark Res. 2025 Oct 14;13:128. doi: 10.1186/s40364-025-00830-x (PMC12522640; doi:10.1186/s40364-025-00830-x)
Supplement: Supplementary file 1 — Supplementary Material 1. [file 40364_2025_830_MOESM1_ESM.pdf]

Supplementary Figure 1

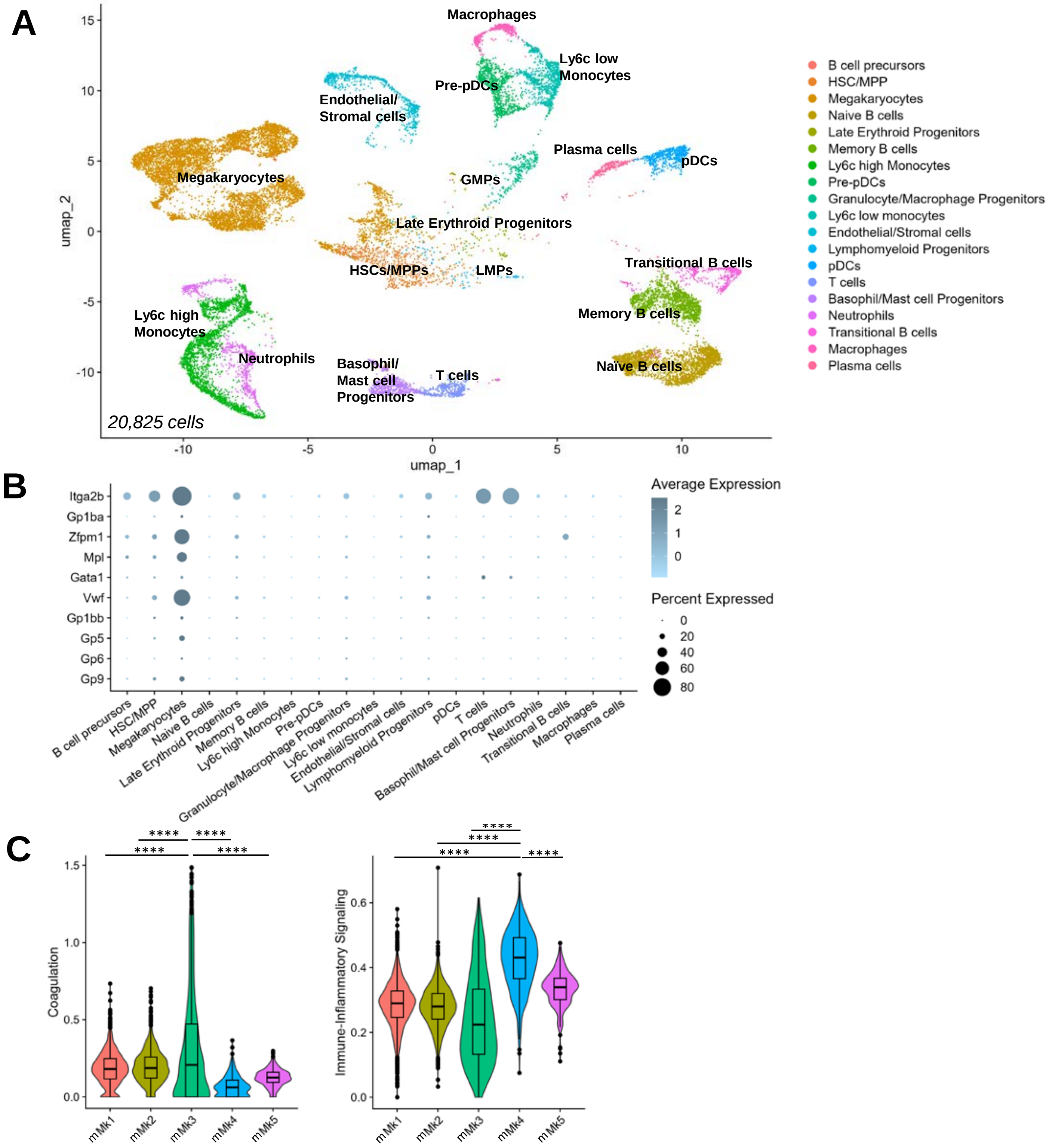
A
Macrophages
Ly6c low Monocytes
Pre-pDCs
Endothelial/ Stromal cells
Megakaryocytes
Plasma cells
pDCs
GMPs
Late Erythroid Progenitors
HSCs/MPPs
LMPs
Transitional B cells
Memory B cells
Ly6c high Monocytes
Neutrophils
Basophil/ Mast cell Progenitors
T cells
Naïve B cells
20,825 cells
umap_1
umap_2
B cell precursors
HSC/MPP
Megakaryocytes
Naive B cells
Late Erythroid Progenitors
Memory B cells
Ly6c high Monocytes
Pre-pDCs
Granulocyte/Macrophage Progenitors
Ly6c low monocytes
Endothelial/Stromal cells
Lymphomyeloid Progenitors
pDCs
T cells
Basophil/Mast cell Progenitors
Neutrophils
Transitional B cells
Macrophages
Plasma cells
B
Itga2b
Gp1ba
Zfpm1
Mpl
Gata1
Vwf
Gp1bb
Gp5
Gp6
Gp9
Average Expression
Percent Expressed
C
Coagulation
Immune-Inflammatory Signaling
mMk1
mMk2
mMk3
mMk4
mMk5
****

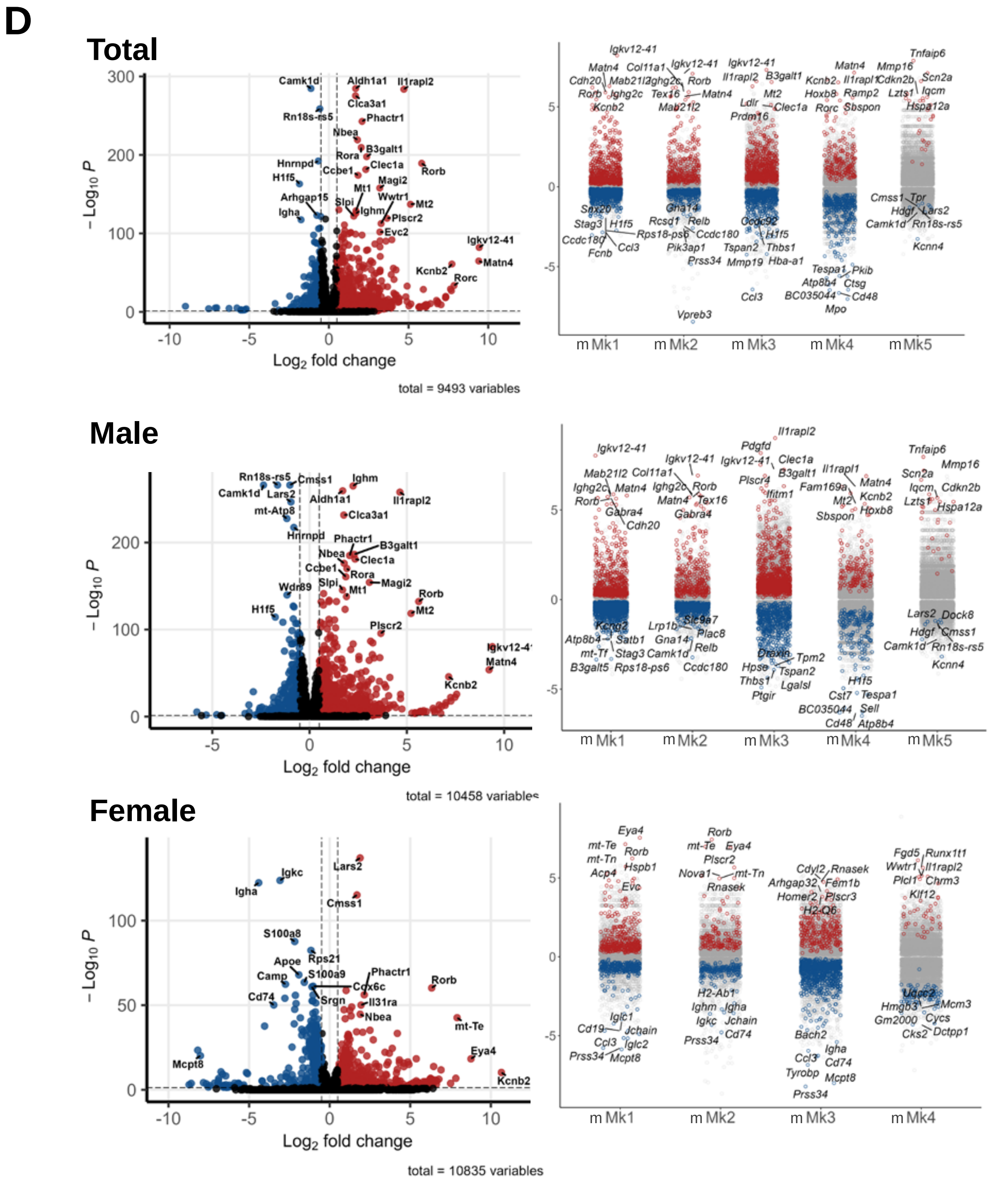

D
Total
Male
Female
Log2 fold change
– Log10 P
total = 9493 variables
total = 10458 variables
total = 10835 variables
m Mk1
m Mk2
m Mk3
m Mk4
m Mk5
Igkv12-41
Matn4
Kcnb2
Rorc
Rorb
Il1rapl2
Aldh1a1
Clca3a1
Phactr1
Camk1d
Rn18s-rs5
Cmss1
Lars2
Igkc
Igha
Eya4
mt-Te

E

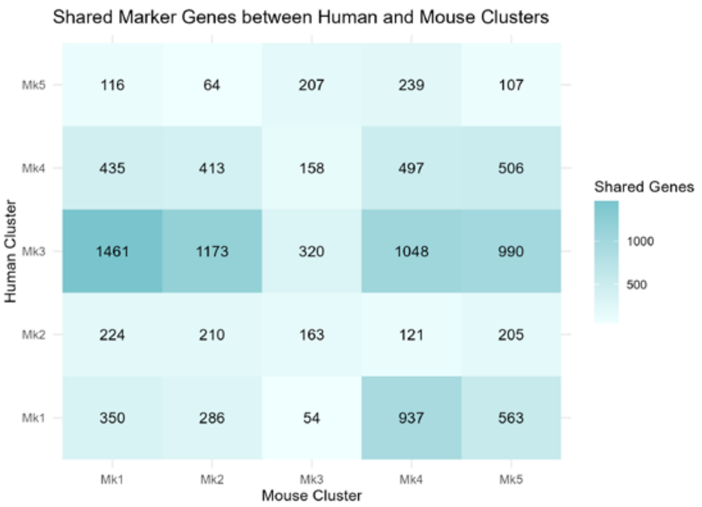
Shared Marker Genes between Human and Mouse Clusters
Human Cluster
Mk5
116
64
207
239
107
Mk4
435
413
158
497
506
Mk3
1461
1173
320
1048
990
Mk2
224
210
163
121
205
Mk1
350
286
54
937
563
Mk1
Mk2
Mk3
Mk4
Mk5
Mouse Cluster
Shared Genes
1000
500

F

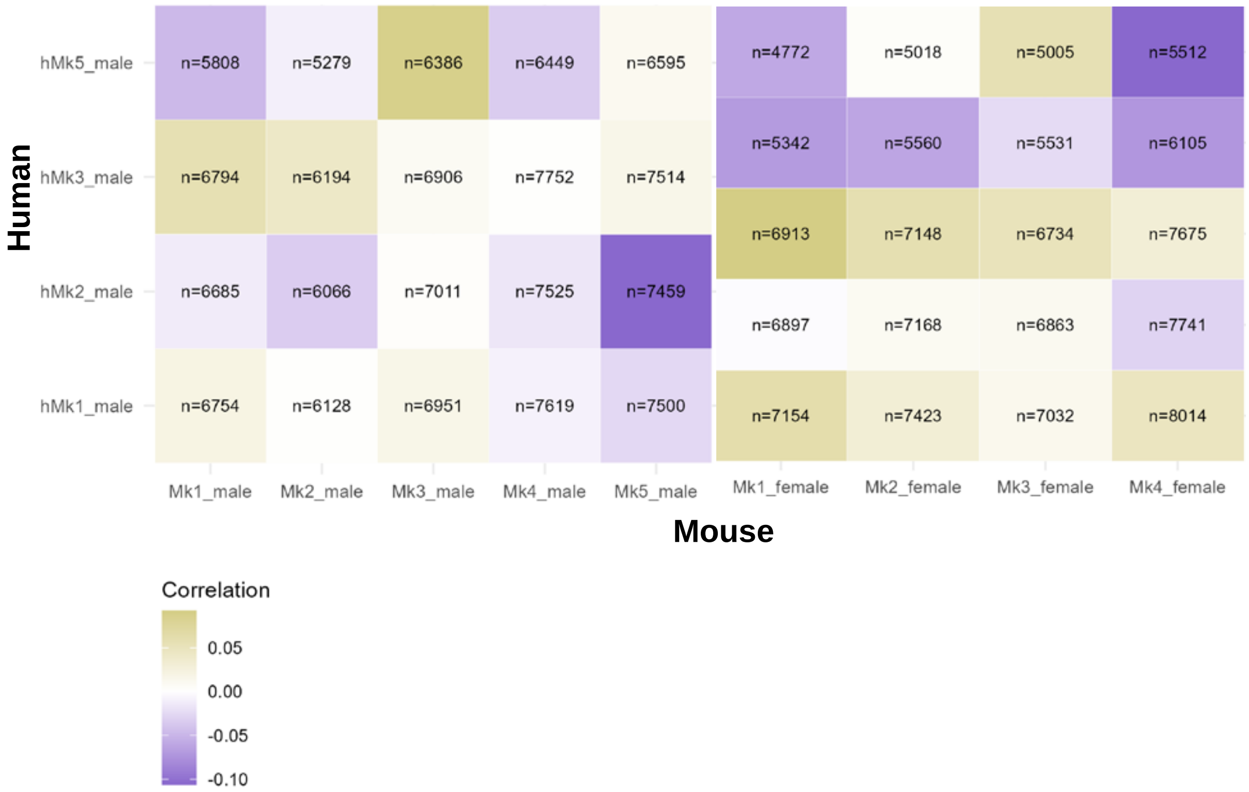
Human
hMk5_male
n=5808
n=5279
n=6386
n=6449
n=6595
n=4772
n=5018
n=5005
n=5512
n=5342
n=5560
n=5531
n=6105
hMk3_male
n=6794
n=6194
n=6906
n=7752
n=7514
n=6913
n=7148
n=6734
n=7675
hMk2_male
n=6685
n=6066
n=7011
n=7525
n=7459
n=6897
n=7168
n=6863
n=7741
hMk1_male
n=6754
n=6128
n=6951
n=7619
n=7500
n=7154
n=7423
n=7032
n=8014
Mk1_male
Mk2_male
Mk3_male
Mk4_male
Mk5_male
Mk1_female
Mk2_female
Mk3_female
Mk4_female
Mouse
Correlation
0.05
0.00
-0.05
-0.10

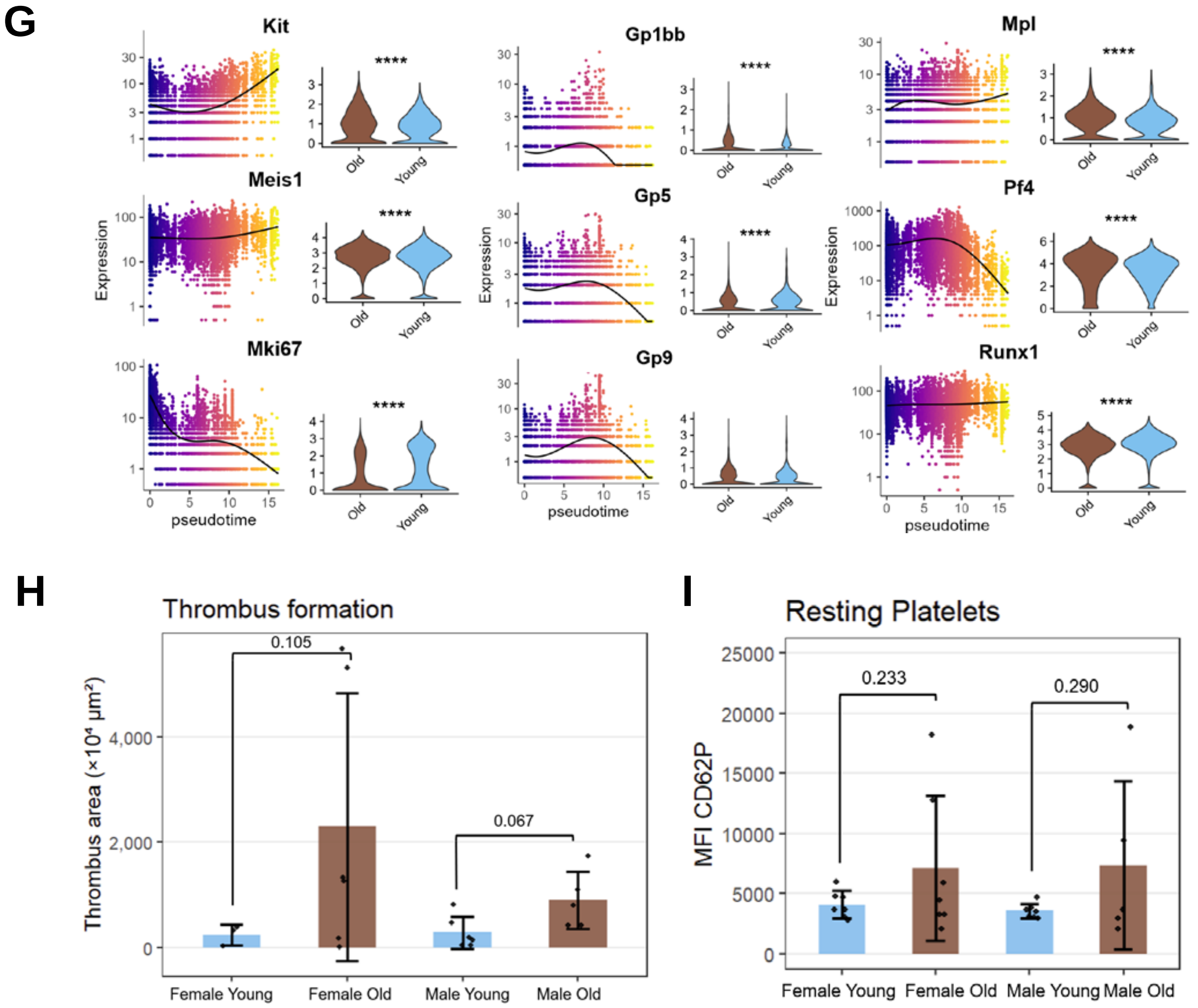
G
Kit
Gp1bb
Mpl
Meis1
Gp5
Pf4
Mki67
Gp9
Runx1
Expression
pseudotime
Old
Young
****
H
Thrombus formation
Thrombus area (×10⁴ μm²)
0.105
0.067
Female Young
Female Old
Male Young
Male Old
I
Resting Platelets
MFI CD62P
0.233
0.290
Female Young
Female Old
Male Young
Male Old

A) UMAP of 20,825 mouse bone marrow cells colored by 20 Louvain clusters (cell types annotated by canonical markers)
B) Dot plot for Mk gene-set scores across subpopulations
C) Violin plot for specific scores in each Mk subpopulation. Kruskal–Wallis followed by Benjamini-Hochberg adjusted pairwise Wilcoxon rank-sum test.
D) Volcano plot of DEGs (|log2FC| > 1, FDR < 0.05) in old vs. young mice. Left Panel = Bulk DEGs; Right Panel = each Mk subpopulation (above or below 1 log2FC and adjusted p-value < 0.05 are color labeled; blue = downregulated, red = upregulated
E) Shared marker genes between human and mouse datasets
F) Composite heatmap displaying the overlap and correlation of DEGs between human and mouse Mk populations. Each tile represents a human–mouse cluster pair, with color indicating the Pearson correlation coefficient of fold changes for shared DEGs and the annotation indicating the number of shared genes (n).
G) Kinetics plot showing the relative expression of representative genes (left). The lines approximate expression along the trajectory using polynomial regressions. Violin plots of gene expression, with Bonferroni adjusted p-values (right).
H) Thrombus formation in whole blood from young and aged mice under high-shear flow (100 dyn/cm^2). Shown are pooled data for the platelet-covered area on the chamber surface at t = 5 min. Bars represent the mean adherent platelet area normalised per square micrometre of chamber surface (expressed as $\times 10^4$ μm^2); error bars indicate ± SD.
I) Platelet activation assay. Fold change of MFI ± IQR of CD62P on untreated washed platelets (Brilliant Violet 421 clone RB40.34).

- *adjusted p-value *<0.05, **<0.01, ***<0.001, **** <0.0001*
- *The lists of genes used to calculate the score are shown in Supplementary Method*
- *Abbreviations: Mk (Megakaryocytes), GMPs (Granulocyte-Macrophage progenitor cells), MPPs (Multipotent Progenitors), HSCs (Hematopoietic Stem cells), LMPs (Lymphomyeloid Progenitors), pDCs (Plasmacytoid Dendritic cells).*

Supplementary Figure 2

A

Group	Age	Sex	Platelet Count (G/L)	Mean Platelet Volume
**Young**	21	F	202	11.6
**Young**	22	M	293	8.5
**Young**	20	F	259	9.4
**Young**	28	M	242	8.8
**Young**	33	F	253	10.4
**Middle-aged**	45	F	299	10.3
**Middle-aged**	40	F	142	11.2
**Middle-aged**	55	M	201	8.5
**Middle-aged**	53	M	198	11.5
**Middle-aged**	41	M	205	11.2

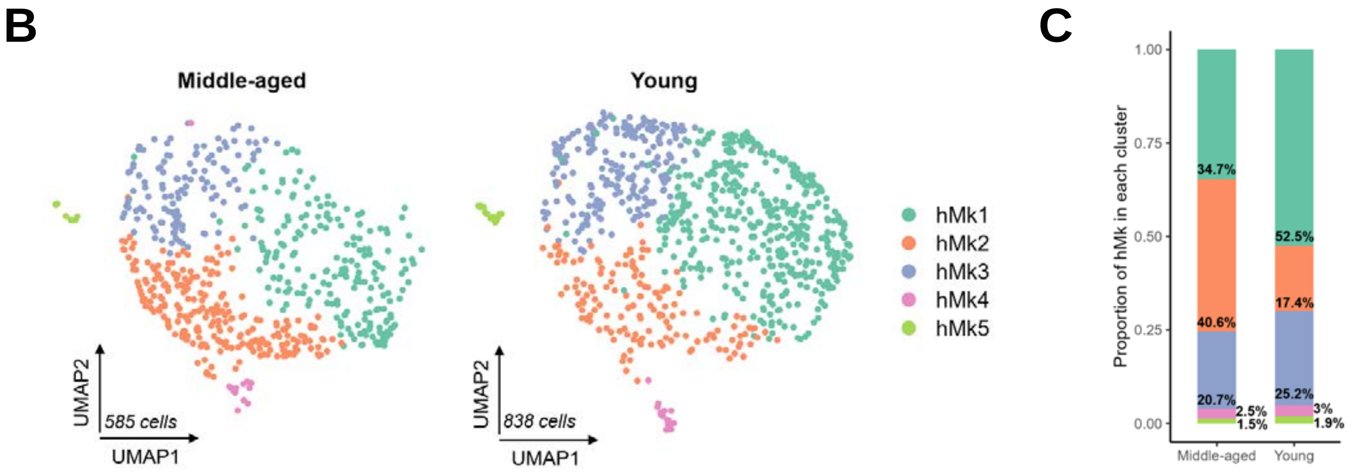

A) Demographic data of human donors and platelet count

B) UMAP of human Mk, colored by subpopulation and separated by age groups

C) Bar graph showing the relative proportion of each Mk subpopulation in young and middle-aged human
