# Supplementary material for: Age- and sex-specific transcriptomic changes drive the prothrombotic potential of megakaryocytes
Source: Biomark Res. 2025 Oct 14;13:128. doi: 10.1186/s40364-025-00830-x (PMC12522640; doi:10.1186/s40364-025-00830-x)
Supplement: Supplementary file 2 — Supplementary Material 2. [file 40364_2025_830_MOESM2_ESM.pdf]

Supplementary methods

Sample preparation and processing

Mice

Young (3-month-old) and old (>24-month-old) C57BL/6 mice (6 females and 5-6 males per group) were purchased from Jackson Laboratory. Animals were housed four per ventilated polycarbonate cage under specific-pathogen-free conditions (22 ± 1'C; 45-55 % humidity; 12 h light/ 12 h dark, lights on 07:00) with ad libitum access to irradiated rodent chow (#D11112201, Research Diets; 19% protein, 61% carbohydrate, 7% fat) and autoclaved tap water.

All animal care and experiments complied with Directive 2010/63/EU and were approved by the University of Zurich's Institutional Animal Care and Use Committee of the Canton Zurich Health Department and Veterinary office (License no. ZH241/19).

<u>Bone marrow collection</u>

Bilateral femora and tibiae were harvested under sterile conditions, freed of soft tissue, rinsed in cold PBS (pH 7.4; Gibco 10010023) + 2 mM EDTA + 0.5% BSA. Bone marrow was isolated by centrifugation at 10000 x g, 20 s, 4'C (Eppendorf 5427 R). Cell suspections were passed through a 40 µm strainer, and erythrocytes were lysed for 2 min at room temperature with ACK Lysis Buffer (Thermo Fisher A1049201) before two PBS washes.

<u>Megakaryocytes enrichment</u>

Nucleated marrow cells (~1 × 10^8) were incubated 20 min on ice with APC-anti-CD41 (clone MWReg30, Invitrogen 17-0411-82, 1:200) and PE-anti-CD42d (clone 2C9.G3, Invitrogen 12-0421-82, 1:100). After washing, cells were labelled (15 min, 4 °C) with anti-PE MicroBeads (Miltenyi 130-105-639) and anti-APC MicroBeads (Miltenyi 130-090-855) and separated on an LD column (Miltenyi 130-042-901). The $CD41^+CD42d^+$ fraction yielded ~1–2 × 10^6 megakaryocytes per mouse.

MACS-enriched cells were sorted on a FACSAria Fusion (BD Biosciences; 488/561/640 nm lasers) using a 100 µm nozzle at 20 psi. Dead cells were excluded with DAPI (1 µg mL^{-1}). Single $CD41^+CD42d^+$ events were collected into 1.5 mL DNA-low-bind tubes containing 100 µL PBS + 0.03 % BSA + RNAlater (1:1 000, Thermo Fisher AM7021). Sorting speed was 25 000–30 000 events per 10 min; post-sort purity was >95 %.

Human

Bone marrow samples from young and middle-aged healthy donors (n=5 per group) were collected at the University Hospital of Zurich. Participants were recruited via advertisements at the University of Zurich and ETH Zurich. Ethical approval was granted by the Cantonal Ethics Commission Zurich (BASEC-Nr. 2021-02204). Informed consent was obtained for all samples.

Biopsy samples were collected in PBS on ice, trimmed off connective tissue, and digested with Collagenase and DNase at 37°C, filtered through a 100 µm strainer, and centrifuged. Cells were labeled with antibodies, sorted by FACS (100 µm nozzle, 4-Way Purity), and collected in FCS-coated tubes containing collection media. Processed cells were resuspended in 50 µl of media, counted with Trypan Blue, and encapsulated according to the 10x Genomics protocol.

Owing to ethical constraints on elective marrow aspiration in adults > 70 years, the present cohort was limited to young and middle-aged donors; nevertheless, an ongoing extension of the study is enrolling 75–85-year-old surgical donors and will integrate CHIP-mutation profiling to assess the impact of advanced age on megakaryocyte biology

Flow cytometry

Platelet activation was assessed by measuring CD62P/P-selectin expression using flow cytometry. Whole blood was anticoagulated, rested at 37°C for 15 minutes and processed to obtain washed platelets.

Washed platelets (100 µL) were stimulated with 0.1 U/mL TRAP6 (Bachem, Cat. No. 4017752) for 20 minutes, then fixed with 4% Paraformaldehyde (PFA) and stored at 4 °C. On the day of analysis, samples were stained with 0.125 µg of anti-CD62P antibody conjugated to BV421 (eBioscience, Cat. No. 46-0626-80) and 0.5 µg of anti-CD41 antibody (BioLegend, Cat. No.133911) for 30 minutes at room temperature. Flow cytometry was performed on a Cytek Aurora system. Platelets were identified based on CD41 positivity and side scatter, with 10,000 events collected per sample. Data were analyzed using FlowJo v10.

Ex vivo thrombosis assay

Microfluidic shear flow assays were conducted using the Bioflux system (Fluxion Biosciences, USA). Channels of a 24-well plate were coated with type I collagen (100 µg/mL) for 1 hour at room temperature, rinsed with PBS containing Ca^{2+}/Mg^{2+}, and blocked with 0.1% BSA for 10 minutes. Calcein AM–labeled whole blood from mice (final concentration 4 µM) was perfused over the collagen-coated surface at shear rates of 100 dyn/cm² for 5 minutes. Platelet adhesion and thrombus formation were imaged every 10 seconds using an inverted EVOS XL microscope (10× objective), and analyzed with Bioflux software, expressed as platelet-covered area (µm²).

Single cell sequencing and Bioinformatics analyses

<u>Library preparation and sequencing</u>

Single-cell suspensions were processed using the 10X Genomics Chromium platform, and libraries were sequenced on an Illumina NovaSeq 6000. Raw BCL files were converted to FASTQ and aligned to the mouse reference genome (mm10) using Cell Ranger v7.2.0 to generate UMI count matrices.

<u>Quality control and data integration</u>

Count matrices for all samples (5–6 individuals per age group) were imported into Seurat v5 in R (2024.04.2+764). Cells were filtered out if they exhibited >15% mitochondrial transcript content, fewer than 500 detected genes, or more than 12,000 detected genes, yielding 20,825 high-quality cells for downstream analysis. To correct for batch effects and integrate across samples, we applied Seurat's standard integration workflow.

<u>Dimensionality reduction and clustering</u>

Normalized data were scaled to regress out unwanted sources of variation (e.g. mitochondrial percentage, cell cycle scores). Principal component analysis (PCA) was performed on the scaled data, and the top PCs were used to compute a two-dimensional UMAP embedding via RunUMAP. Unsupervised clustering was carried out using FindClusters (Louvain algorithm) on the same PC space. Cluster identities were assigned by running FindAllMarkers to extract the top differentially expressed genes per cluster; clusters were named based on the top 50 marker genes.

Module scoring and signature analysis

Megakaryocyte (Mk) cells were scored for predefined functional gene signatures using AddModuleScore. Signature gene lists were curated from Gene Ontology and literature sources (see below).

Differential gene expression and enrichment

Within each cluster, differential expression between young and old cells was assessed using Seurat's FindMarkers with a two-sided Wilcoxon rank-sum test (default parameters), focusing on highly variable genes. Resulting gene lists were subjected to GO Biological Process 2023 enrichment analysis via EnrichR; terms with adjusted $P < 0.05$ were considered significant.

Trajectory inference

To model Mk maturation, we subsetted Mk clusters and applied Monocle3. After batch correction via mutual nearest neighbor alignment, Monocle3 constructed a pseudotemporal trajectory in two dimensions. The trajectory root was defined by the node most enriched for young cells. Stage-dependent gene expression along pseudotime was evaluated using Moran's I test, identifying 2,112 trajectory-dependent genes ($q = 0$). These genes were partitioned into three co-expression modules. Each module underwent GO enrichment (EnrichR, GO Biological Process 2023), retaining terms with adjusted $P < 0.05$; the top ten terms per module are visualized in Figure 2D.

Subpopulation frequency analysis

We report subpopulation frequencies on pooled single-cell data from all 5–6 individuals per group: cells were combined prior to clustering, and proportions were calculated on the aggregate dataset to maximize cell-level resolution and standardize analyses across samples.

Statistical analyses

All statistics and data visualizations were performed in R (2024.04.2+764) using ggplot2. Depending on data type, comparisons employed two-tailed Student's t-tests, one-way ANOVA, or Chi-square tests. Wilcoxon rank-sum tests were used for module-score comparisons. Multiple testing corrections were applied using the Benjamini–Hochberg false discovery rate; Bonferroni adjustment was used for post hoc pairwise comparisons.

Lists of genes used to calculate module scores

Supplementary Fig. 1C

Coagulation

Blood Coagulation, Fibrin Clot Formation (GO:0072378)

FGB, FGG, F13A1, F12, GP1BB, GP5, GP1BA, FBLN1, GP9, F8, THBD, FLNA, F13B, FGA

Blood Coagulation, Intrinsic Pathway (GO:0007597)

F8, F12, FLNA, GP1BB, GP5, GP1BA, GP9

Regulation Of Fibrinolysis (GO:0051917)

F11, SERPINE1, SERPINF2, F12, CEL, NRG1, THBS1, PLAU, PLAUR, PLAT, PLG, F2, VTN, USF1, THBD, KLKB1

Immune/Inflammatory response

Immune-Inflammatory response (GO:0002696, Ref.1-2)

Lsp1 Cd53 CEBPA IL33 TSLP KARS1 WNT5A EHHADH THBS1 IRGM HSPD1
PLPP6 CLEC4D TNIP2 CLEC7A SPACA3 CD226 TLR6 HAVCR1 TLR4
CRLF2 NECTIN2 Cd48 Ccl3 Spi1 S100a11 Tnfaip3 thbs1 Zfp36 Slamf1 Alox5
Cd74 Slpi Dst Mapkapk2 Cd44 Serpini1 S100a10 Txnip Tox Prdm16
Clnk C1qtnf7 Tgfbr3 Ifitm1 Jund Casp12 Il20ra Cd24a Nfkbia Litaf Txndc11 Alcam
Selp F11r Acss2 Slpi Nkg7 Nrgn Ctla2a Anxa1 Jund Tlr4

Figure 1H

Cholesterol biosynthesis & Transport

Cholesterol Biosynthetic Process (GO:0006695)

G6PD CYP51A1 HMGCS1 LSS DHCR24 ACLY PLPP6 GLB1 MVD DHCR7
MVK CES1 INSIG2 INSIG1 HMGCR MSMO1 HSD17B7 TM7SF2 NSDHL
NPC1L1 PMVK ARV1 SC5D LBR FDFT1

Cholesterol Transport (GO:0030301)

STARD3 ABCG8 STARD4 STARD5 CAV1 APOA2 APOA5 APOA4 NPC1
NPC2 SOAT2 TSPO2 APOA1 SOAT1 RELCH ABCG1 ABCG4 ABCG5 CES1 GUCY2C VPS4A CLU
LCAT STX12 NPC1L1 SERAC1 CD36 LDLR MSR1 STARD3NL OSBPL5 ABCA7
ABCA5 AKR1C1 CEL OSBPL2 ABCA8 LIMA1 APOC1 APOC3 APOC2 CETP LIPC
TSKU LIPG APOM OSBP APOE LDLRAP1 SCARB1 APOB
