# Supplementary material for: Age- and sex-specific transcriptomic changes drive the prothrombotic potential of megakaryocytes
Source: Biomark Res. 2025 Oct 14;13:128. doi: 10.1186/s40364-025-00830-x (PMC12522640; doi:10.1186/s40364-025-00830-x)
Supplement: Supplementary file 4 — Supplementary Material 4. [file 40364_2025_830_MOESM4_ESM.pdf]

# Supplementary Table 2: Lists of DEGs genes according to function in male and female human donors

| | Female | Male |
|---|---|---|
| **Mitochondrial-related genes** | | |
| ETC Complex I | ↑MT-ND1, MT-ND2, MT-ND3, MT-ND4, ↑MT-ND4L<br>↑NDUFA1, NDUFA3, NDUFB1, NDUFS5 | ↓MT-ND1, MT-ND2, MT-ND3, MT-ND4, ↓MT-ND5<br>↑NDUFA1 |
| ETC Complex III | ↑MT-CYB / ↑UQCR11, UQCRB | ↓MT-CYB / ↑UQCR11 |
| ETC Complex IV | ↑MT-CO1, MT-CO2, MT-CO3 / ↑PET100<br>↑COX6C, COX7C | ↓MT-CO1, MT-CO2, MT-CO3<br>↑COX6B1 |
| ETC Complex V | ↑MT-ATP6, MT-ATP8 / ↓ATP5F1C, ↑ATP5MC3<br>↑ATP5ME, ATP5MF, ATP5MG, ATP5F1E | ↓MT-ATP6, MT-ATP8<br>↑ATP5MPL, ATP5MC2, ATP5MC3 |
| Mt RNAs and tRNAs | ↑MT-TT, MT-TS2, MT-TV, MT-TL1<br>↑MT-RNR2 | ↓MT-RNR1, MT-RNR2 |
| Other Mt factors | ↑TIMM10, TOMM7, ROMO1, MTLN, ↑MTRNR2L1 / ↓CHCHD3 | ↑MRPS21, MRPL52, MTRNR2L1 |
| **Thromboinflammation-related genes** | | |
| Innate immune response | ↑P2RY14 / ↓CLEC11A | ↑GPR171, CD164 / ↓P2RY14 |
| Adaptive immune response | ↑CD69, CD81, HLA-B,<br>↓HLA-DRB5, IGKC, IGLC2, ITGA4 | ↑HLA-DRB5, HLA-DQA2<br>↓IGKC, PRKCQ, RUNX1 |
| Inflammation | | ↑CXCL2 / ↓CXCL12, JAK1 |
| Platelet | ↑PF4, GP5*, GP9*, GP6*, ITGA2B*, ITGA2*, HTR2A* | |

*All genes listed except * have adjusted p-value < 0.05 using Benjamini and Hochberg method; Genes with * have p-value < 0.05*
*↑Upregulated, ↓Downregulated*
